# A novel polypeptide-modified fluorescent gold nanoclusters for copper ion detection

**DOI:** 10.1038/s41598-022-10500-9

**Published:** 2022-04-22

**Authors:** Hong Zhuang, Xinyu Jiang, Sijia Wu, Shujin Wang, Yong Pang, Yanjun Huang, Haiyang Yan

**Affiliations:** College of Food Science and Engineering, Jilin University, No. 5333 Xi’an Road, Changchun, 130062 China

**Keywords:** Assay systems, Analytical chemistry

## Abstract

Biomolecule-functionalized fluorescent gold nanocluster (AuNCs) have attracted a lot of attention due to good biocompatibility, stable physicochemical properties and considerable cost advantages. Inappropriate concentration of Cu^2+^ may cause a variety of diseases. In this study, AuNCs were synthesized in alkaline aqueous solution using bovine serum albumin (BSA) as a template. And then, the peptide CCYWDAHRDY was coupled to AuNCs. Furthermore, the fluorescence of synthesized CCYWDAHRDY-AuNCs response to Cu^2+^ was evaluated. As the results shown that the CCYWDAHRDY-AuNCs can sensitively detect Cu^2+^. After adding Cu^2+^ to the probe system, the fluorescence of the CCYWDAHRDY-AuNCs was quenched. The detection conditions were at pH 6 and 30 °C for 10 min, the linear relationship between Cu^2+^ concentration and fluorescence intensity were good in the range of 0.1 ~ 4.2 μmol/L. The regression equation was y = − 105.9x + 693.68, the linear correlation coefficient is 0.997, and the minimum detection limit was 52 nmol/L.

## Introduction

The accumulation of heavy metal ions in the environmental system increases the risk of harm to the environment and human health^1–5^. Heavy metal ions can easily interfere with enzymes and nucleic acids and change the biological activities of organisms^6^. Cu^2+^ plays an important role in biology as a transition metal, and proper intake of Cu^2+^ is necessary to maintain the health of the organism^7,8^. However, an inappropriate concentration of Cu^2+^ may cause a variety of diseases. For example, anemia and decreased vision are symptoms caused by the lack of Cu^2+^, and excessive Cu^2+^ content may accelerate the deterioration of Alzheimer's disease and Parkinson's disease^9–13^. Cu^2 +^ are widely distributed in soil and water, which was easily enter the human body through food chain. Real-time monitoring of Cu^2+^ is a prerequisite for food safety and disease prevention^14,15^. Fluorescence spectroscopy, colorimetry, electrochemical analysis and gas chromatography have been applied to detection of Cu^2+^^16–20^. Fluorescence analysis technology has attracted widespread attention due to its high sensitivity, easy operation and fast detection speed. With the development of nanomaterials and fluorescent probes, gold nanocluster (AuNCs) as fluorescent sensors for detecting pollutants in the environment and food, have attracted the attention of many researchers^21,22^.

AuNCs are composed of dozens or even hundreds of gold atoms, with a mean particle size of less than 2 nm^23,24^. Compared with traditional fluorescent dyes or proteins, AuNCs have excellent properties such as little effect on the activity of organisms, high stability, low toxicity and high biocompatibility due to their chemical inertness and ultra-fine size^25^. In addition, AuNCs have a larger stokes shift and stronger fluorescence emission^26^. With the addition of multivalent metal cations, the Au–S bond on the AuNCs surface is broken due to the interaction of carboxyl groups and metal ions, resulting in luminescence quenching^27,28^.

The fluorescence properties of AuNCs can be adjusted by using appropriate ligands and biocompatible scaffolds^29,30^. Previous studies have shown AuNCs could be prepared using proteins, amino acids, peptides, thiols, nucleic acids and other biomolecules as ligands, which have a high degree of biocompatibility and can be used for interference-free detection of biological materials^31–34^. Particularly, peptides are often used to synthesize biocompatible and functional metal nanoclusters due to its special three-dimensional structure, adjustable sequence, convenient synthesis and economical price^33^. For instance, Yuan and co-workers compared Au_25_ NCs protected by GSH long-chain peptide nucleic acid with electron rich -COOH and -NH2 groups produced stronger luminescence^35^. Cysteine (C) has a good coordination ability^36^, and tyrosine (Y) has a strong ability to reduce metal ions^37^, C and Y are usually introduced into the peptide sequence to prepare AuNCs. Certain peptides can be coupled with AuNCs to quickly and effectively detect highly toxic ions. For example, the CCYR_6_H_4_-AuNCs bioluminescence sensor reduces the detection limit and improves the selectivity to Hg^2+^ in water^38^.

Fluorescent probes can be applied to the assay of Cu^2+^ due to the fluorescence quenching behavior of Cu^2+^. In this study, the novel fluorescent probes of CCYWDAHRDY-AuNCs were synthesized for detecting intracellular Cu^2+^ in the water. First, BSA was used as a reducing agent and stabilizer to prepare AuNCs, and then CCYWDAHRDY solution and AuNCs were stirred and incubated at 25 °C for 24 h to obtain CCYWDAHRDY-AuNCs. Moreover, the specificity of CCYWDAHRDY-AuNCs responses to Cu^2+^ was evaluated.

## Materials and methods

### Materials

All metallic ions (*i.e.* Cu^2+^, Pb^2+^, Zn^2+^, Ni^2+^, and potassium) were purchased from Sigma (St Louis, MO, USA). Chloroauric acid (AuCl_3_·HCl·4H_2_O), Sodium hydrogen phosphate (Na_2_HPO_4_) and Sodium dihydrogen phosphate (NaH_2_PO_4_) were obtained from Sinopharm Chemical Reagent Company (Shanghai, China). Bovine serum albumin (BSA) was bought from Changchun Dingguo Reagent Co., Ltd. (Jilin, China). Peptide CCYWDAHRDY was purchased from GL Biochem (Shanghai) Ltd (Shanghai, China). All chemicals were analytical reagent grade and used directly without further purification. Distilled water was used throughout the experiment.

### Synthesis of CCYWDAHRDY -AuNCs

#### Preparation of AuNCs

All glasswares were cleaned in freshly prepared aqua regia solution (HCl: HNO_3_ volume ratio = 3:1) and thoroughly rinsed in distilled water before use. First, 5 mL of 10 mmol/L HAuCl_4_ aqueous solution and 5 mL of 50 mg/mL BSA solution were mixed under stirring at 37 ℃ for 5 min. Next, 1 mL of 1 mol/L NaOH was added to the above mixtures. And the mixture was stirred at 37 ℃ for 24 h to obtain the AuNCs crude product. Furthermore, the AuNCs crude product was dialyzed in distilled water to remove the excess of large-particles to obtain AuNCs.

#### Preparation of CCYWDAHRDY -AuNCs

The peptide CCYWDAHRDY designed in our study was synthesized by the solid phase procedure using the FMOC protected amino acids synthesis methods^39^. The synthesis of CCYWDAHRDY -AuNCs was performed by the method described by our previous study^40^. First, CCYWDAHRDY powder was dissolved in ultrapure water to obtain 1 mg/mL CCYWDAHRDY aqueous solution. Secondly, 0.5 mL of CCYWDAHRDY aqueous solution was added into 2 mL AuNCs solution. The above mixture was stirred at 25 ℃ for 24 h gently to obtain CCYWDAHRDY-AuNCs solution, which was stored at 4 ℃ in the dark.

### Characterization of CCYWDAHRDY-AuNCs

The fluorescence intensity of AuNCs and CCYWDAHRDY-AuNCs were measured using RF5301 fluorescence spectrophotometer (Shimadzu Enterprise Management (China) Co., Ltd.). The shape and size of AuNCs and CCYWDAHRDY-AuNCs were analyzed using FEI Titan ETEM G2 transmission electron microscope (Shanghai Zhengfei Electronic Technology Co. Ltd.). And the ultraviolet absorption spectrum was measured using UV1800 UV–Visible spectrophotometer (Shanghai Precision Instrument Co. Ltd.).

### Detection conditions of the fluorescent probes of CCYWDAHRDY-AuNCs

#### Optimization of pH value

CCYWDAHRDY-AuNCs solution of 0.1 mL and phosphate buffered saline (PBS) solution of 0.84 mL with different pH values, *i.e.,* 4, 5, 6, 7 and 8 were mixed, next 0.06 mL of 60 μmol/L Cu^2+^ standard solution was added. The fluorescence intensity of the mixture was subsequently measured. In the control group, Cu^2+^ standard solution was replaced by PBS solution, and the fluorescence intensity of the mixture was subsequently measured.

#### Optimization of reaction temperature

CCYWDAHRDY-AuNCs solution of 0.1 mL and phosphate buffered saline (PBS) solution 0.84 mL of were mixed, next 0.06 mL of 60 μmol/L Cu^2+^ standard solution was added. Then, the fluorescence intensity of the mixture was subsequently measured at different temperature (*i.e.,*10, 20, 30, 40 and 50 ℃). In the control group, Cu^2+^ standard solution was replaced by PBS solution, and the fluorescence intensity of the mixture was subsequently measured.

#### Optimization of reaction time

CCYWDAHRDY-AuNCs solution of 0.1 mL and phosphate buffered saline (PBS) solution of 0.84 mL were mixed, next 0.06 mL of 60 μmol/L Cu^2+^ standard solution was added, then, the fluorescence intensity of the mixture with different reaction time (*i.e.,* 0, 5, 10, 15, 20, 25 and 30 min) was subsequently measured. In the control group, Cu^2+^ standard solution was replaced by PBS solution, and the fluorescence intensity of the mixture was subsequently measured.

### Fluorescence detection of the CCYWDAHRDY-AuNCs to Cu^2+^

CCYWDAHRDY-AuNCs (100 μL) were mixed with 0.06 mL of different concentrations of Cu^2+^ (*i.e.,* 0.6, 1.2, 1.8, 2.4, 3.0, 3.6 and 4.2 μmol/L) in PBS buffer (pH = 6), the final volume of the reaction system is 1 mL. The mixture was incubated at 30 ℃ for 10 min. Then, spectral scanning was performed and recorded on a fluorescence spectrophotometer. The detection curve of the Cu^2+^ concentration was established using the fluorescence efficiency (F_0_/F) as the ordinate. F_0_ and F respectively indicated the maximum fluorescence intensity of the solution system in the absence and presence of the Cu^2+^. Fluorescence intensity of AuNCs with Cu^2+^ was also recorded.

### Selectivity experiments

The fluorescence intensities of test solution containing Cu^2+^ with different concentrations of interferences were measured. The following metal ions were used: Co^2+^, Fe^3+^, Ni^2+^, Zn^2+^, Ca^2+^, K^+^, Na^+^, Pb^2+^.

### Statistical Analysis

Data were expressed as means ± SD (n = 3) and the differences were carried out by means of one-way ANOVA test followed by Least Significant Difference (LSD) test using SPSS (SPSS Inc., Chicago, IL, USA).

## Results and discussion

### Characterization of the AuNCs and CCYWDAHRDY-AuNCs

**Figure 1:**
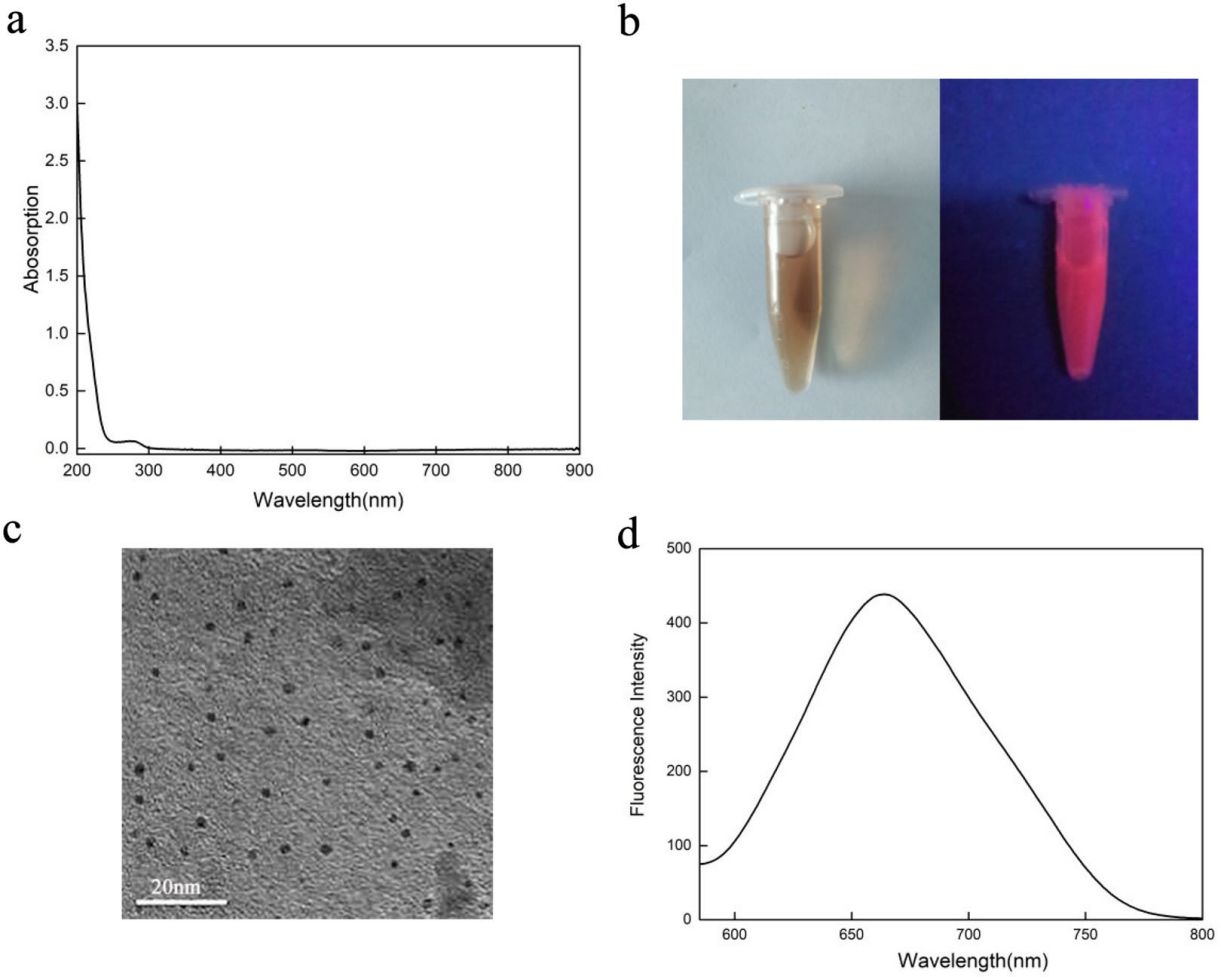
Characterization of the AuNCs: (**a**) UV–visible absorption spectrum of the AuNCs, (**b**) The photographs of the AuNCs (The photograph on the left is in daylight, The photograph on the right shows a 350 nm UV lamp), (**c**) The TEM photograph of the AuNCs, (**d**) Fluorescence emission spectra of AuNCs at an excitation wavelength of 260 nm.

BSA was used as reducing agent for the synthesis reaction and protective agent for the cluster. As shown in Fig. 1(a), the curve did not exhibit the characteristic absorption peak around 520 nm of AuNCs, thus there was no nanocrystals produced during the synthesis of the AuNCs, which indicated that the AuNCs had a small particle size and well dispersed. As shown in Fig. 1(b), the synthesized AuNCs were light brown/yellow under visible light and emitted an intense orange fluorescence under the illumination of a 350 nm UV lamp. the average particle size of the AuNCs was about 1.8 nm with a good dispersion and no particle agglomeration [shown in Fig. 1(c)], which was consistent with previous reports^41,42^.

As shown in Fig. 1(d), and the maximum emission wavelength of AuNCs was 650 nm. BSA modified AuNCs had Au_0_-Au_1_ core–shell nanostructures and produced fluorescence was the charge transfer between the fluorescent ligands and the Au^+^. The tyrosine residue in BSA had the ability to reduce Au^+^ to Au under alkaline conditions. At the same time, the cysteine residue in BSA could capture the AuNCs in the system through the Au–S bond, and BSA increased the stability of the reaction system.

**Figure 2:**
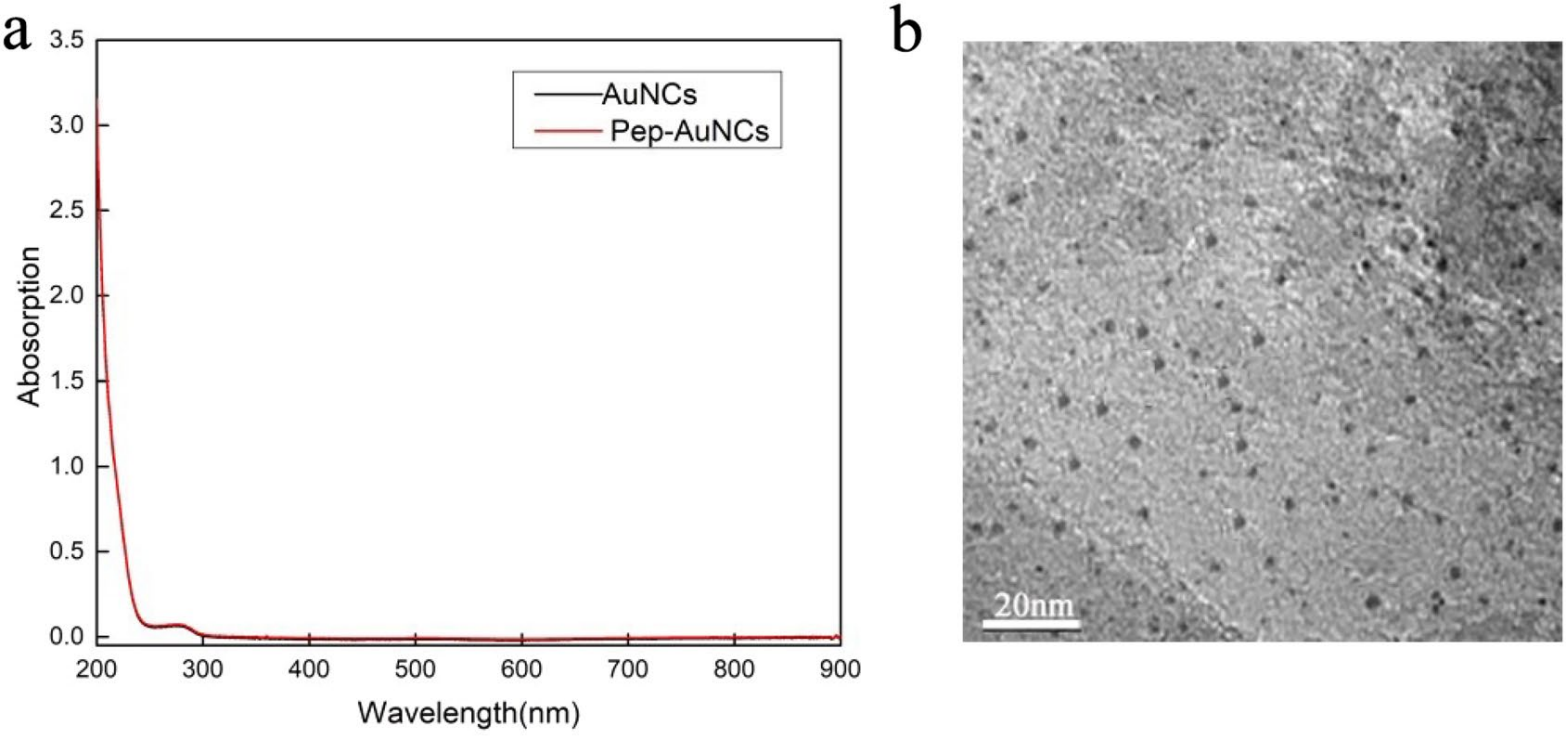
Characterization of the CCYWDAHRDY-AuNCs: (**a**) TEM photograph of the CCYWDAHRDY-AuNCs, (**b**) UV–visible absorption spectrum of the AuNCsand the CCYWDAHRDY-AuNCs.

**Figure 3:**
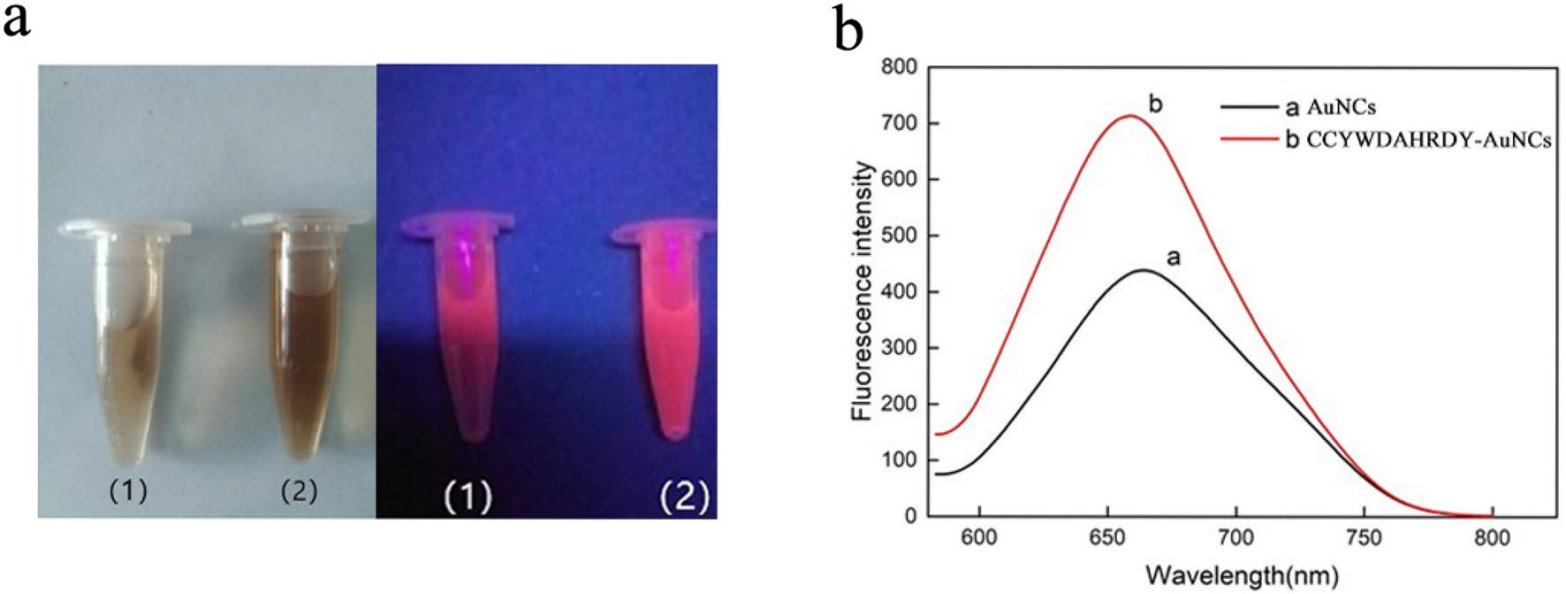
The fluorescence intensity of AuNCs and CCYWDAHRDY-AuNCs: (**a**) Photographs of the AuNCs (1) and the CCYWDAHRDY-AuNCs (2) in daylight (left) and under a 350 nm UV lamp (right), (**b**) Fluorescence emission spectra of the AuNCs and the CCYWDAHRDY-AuNCs at an excitation wavelength of 260 nm.

Figure 2(a) showed that the dispersibility of the system was unchanged when the CCYWDAHRDY was coupled with the AuNCs. There was no obvious change in the particle size and no aggregation occurred, which suggested the system will have a strong fluorescence emission and stable properties. UV–vis absorption spectra were used to investigate the optical characterization and structure of AuNCs and CCYWDAHRDY-AuNCs. As shown in Fig. 2(b), the spectra of the AuNCs was unchanged after the coupling with the CCYWDAHRDY. The CCYWDAHRDY used in our experiments successfully modified the AuNCs without affecting AuNCs properties^38^. It was observed from Fig. 3(a) that the AuNCs coupled with the CCYWDAHRDY were slightly darker than the AuNCs under natural light, whereas the orange-red fluorescence emission of the CCYWDAHRDY -AuNCs under ultraviolet light was mostly similar to that of the AuNCs.

The fluorescence of the CCYWDAHRDY-AuNCs was compared to that of AuNCs. As shown in Fig. 3(b), the fluorescence of the AuNCs increased significantly after coupling the CCYWDAHRDY. That maybe CCYWDAHRDY contained a functional tripeptide chain CCY, where the phenolic group in the tyrosine could reduce trivalent gold ions to gold atoms, and the cysteine could capture the AuNCs so that the CCYWDAHRDY could bind the AuNCs. Moreover, the electron-rich oxygen atom or the nitrogen atom in the CCYWDAHRDY and the functional group (carboxyl group and amino group) in the ligand could effectively enhance the electron transfer, thereby increasing the fluorescence intensity of the AuNCs modified by the CCYWDAHRDY. The tryptophan (W) in CCYWDAHRDY had a strong reducing ability, which could promote the formation of AuNCs and increase the fluorescence intensity. At the same time, the CCYWDAHRDY acted as a suitable stabilizer and further protected the fluorescence of the AuNCs thereby avoiding the agglomeration of AuNCs into larger particles induced by external environmental factors and enhanced the fluorescence stability of the AuNCs.

### Optimization of test conditions

**Figure 4:**
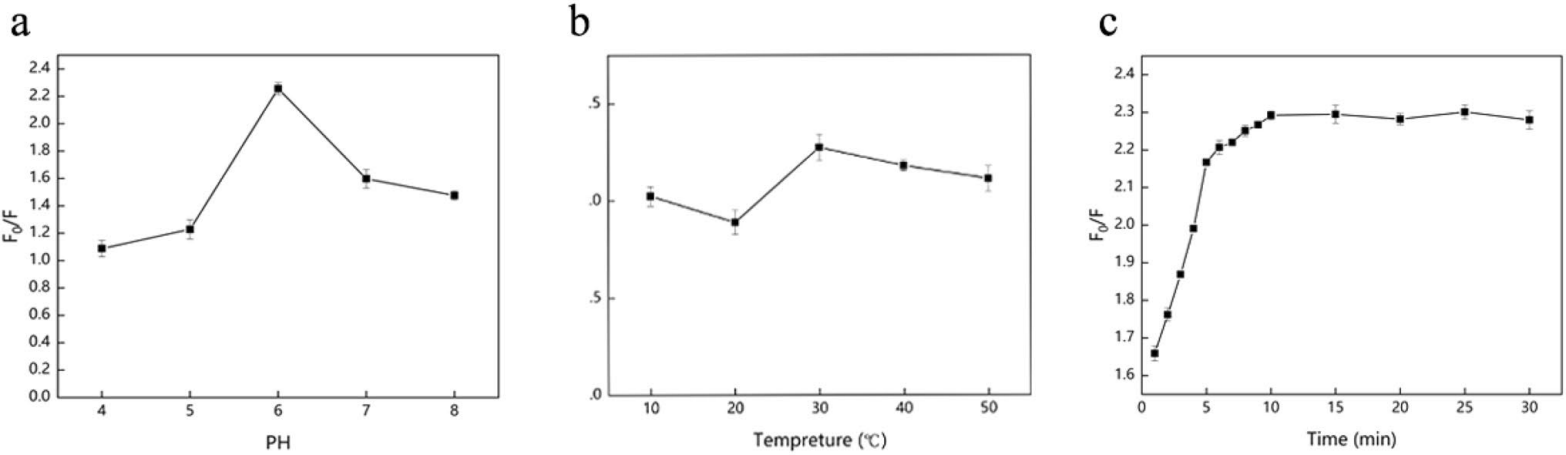
Influence of different environmental factors on the effect of Cu^2+^ quenching CCYWDAHRDY-AuNCs Fluorescence: (**a**) Fluorescence-emission intensity of CCYWDAHRDY-AuNCs to Cu^2+^ at different pH values, (**b**) Fluorescence-emission intensity of the CCYWDAHRDY-AuNCs to Cu^2+^ at different temperatures, (**c**) Evolution of the intensity of the fluorescence emission of CCYWDAHRDY-AuNCs to Cu^2+^ over time.

In order to select the best experimental conditions, the main factors include pH, temperature and reaction time. 650 nm excitation wavelength and 60 μmol/L Cu^2+^ standard solution was used by us to detect the best reaction condition. The effects of the different pH values on the fluorescence response of the CCYWDAHRDY-AuNCs were studied and the pH of the experimental system was optimized, as shown in Fig. 4(a). When the pH of the system was 6, the fluorescence intensity ratio F_0_/F was the highest. When the pH increased, F_0_/F became stable and slightly decreased. Therefore, PBS buffer at pH 6.0 was choose as the optimal detection condition.

The temperature played a dominant role in the fluorescence quenching system. The effect of the temperature on the detection was investigated. As shown in Fig. 4(b), when the temperature raised from 10 to 30 °C, the fluorescence intensity ratio F_0_/F gradually increased, and the fluorescence intensity ratio F_0_/F reached a maximum at 30 °C. When the temperature continued to rise, the quenching ratio gradually decreased. Therefore, 30 °C was the optimum detection temperature.

The fluorescence quenching of CCYWDAHRDY-AuNCs by Cu^2+^ was studied as a function of the reaction time (Fig. 4c). The fluorescence intensity of the reaction decreased rapidly within 0 ~ 5 min. The fluorescence intensity decreased over time. After 10 min, the fluorescence remained relatively stable and did not decrease significantly. Therefore, 10 min was considered to be the optimal reaction time.

### Linear relationship and sensitivity for the detection of Cu^2+^ using CCYWDAHRDY -AuNCs

**Figure 5:**
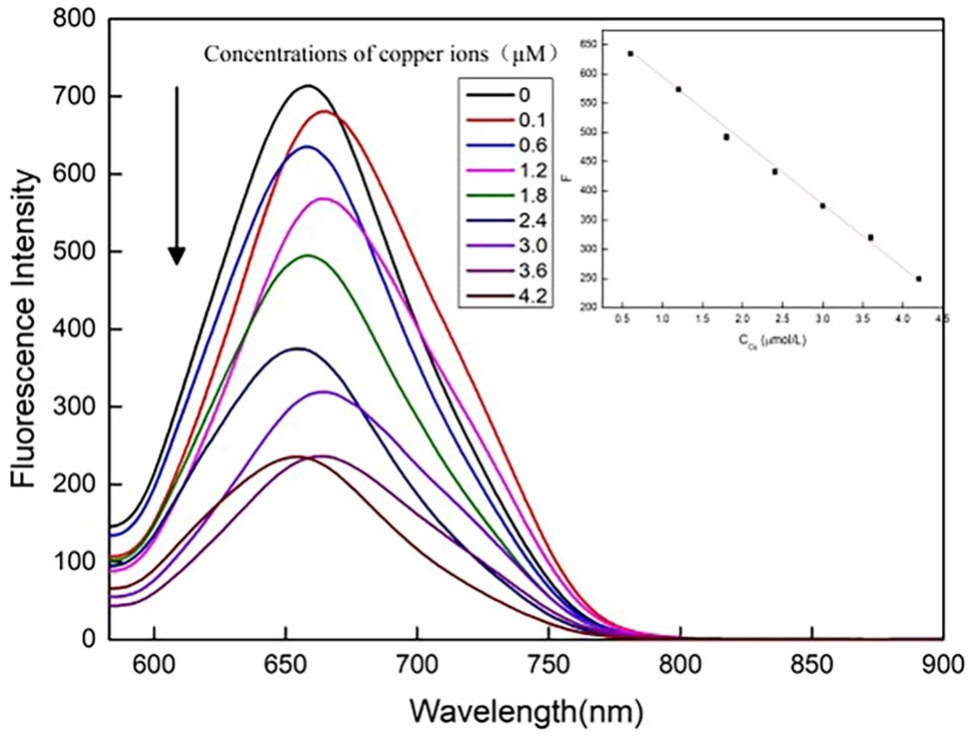
Fluorescence response of the CCYWDAHRDY-AuNCs to the different Cu^2+^ concentrations.

The successful coupling of CCYWDAHRDY and AuNCs could achieve highly sensitive monitoring of Cu^2+^. The tripeptide sequence DHA could orbitally overlap with Cu^2+^ through nitrogen atoms to form a stable planar structure, which could achieve the purpose of identifying Cu^2+^. The CCYWDAHRDY-AuNCs under the optimal reaction conditions were used to quantitatively detect Cu^2+^. As shown in Fig. 5, for a range of Cu^2+^ concentrations within 0.1 ~ 4.2 μmol/L, the fluorescence intensity of the CCYWDAHRDY-AuNCs and F_0_/F gradually decrease when the concentration of Cu^2+^ added to the CCYWDAHRDY-AuNCs fluorescence system increases. There is a linear correlation between F_0_/F and the Cu^2+^ concentrations. The linear regression equation was y = − 105.9x + 693.68 with a correlation coefficient of 0.997. The minimum detection limit for S/N = 3 was 52 nmol/L. As shown in Table 1, compared to previous studies, the detection limit of assay for Cu^2+^ detected by CCYWDAHRDY-AuNCs was lower. It is also lower than the maximum allowable concentration of Cu ^2+^ in drinking water set by the World Health Organization (WHO) and the United States Environmental Protection Agency (EPA) (20 and 30 μmol/L, respectively). Generally, CCYWDAHRDY-AuNCs will have broad application prospects for determination of Cu^2+^.

**Table 1:** comparson with other detection methods.

Method	LOD (nmol∙L^-1^)	Linear range (μmol∙L^-1^)	References
LRL—AuNCs two-photon fluorescent probe	3900	5–80	^43^
Riboflavin-AuNCs probe	900	0–30	^44^
Q1OEt-modified SiNWs sensor	380	2–20	^45^
Silicon quantum dots	500	50–10,000	^46^
CCYWDAHRDY-AuNCs fluorescence sensor	52	0.1–4.2	This work

### Comparison of the CCYWDAHRDY-AuNCs and the AuNCs for the detection of Cu^2+^

**Figure 6:**
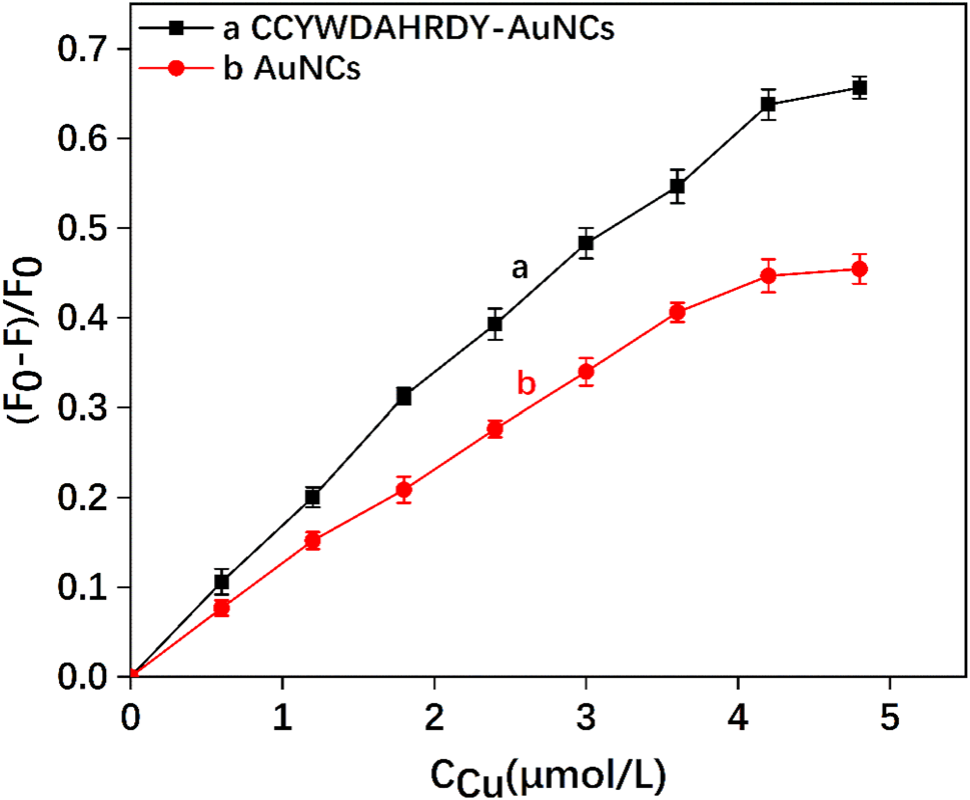
Fluorescence response of the AuNCs and CCYWDAHRDY-AuNCsand the to the different concentrations of Cu^2+^.

As shown in Fig. 6, the slope of response curve of the CCYWDAHRDY-AuNCs to the concentration of Cu^2+^ was larger than that of the AuNCs, which indicated CCYWDAHRDY-AuNCs had a higher sensitivity. The tripeptide sequence DAH could form a stable planar structure with the Cu^2+^. Therefore, in the entire fluorescence detection system, CCYWDAHRDY-AuNCs can more sensitivity recognize Cu^2+^.

### Selectivity of the CCYWDAHRDY–AuNCs detector for detecting Cu^2+^

**Figure 7:**
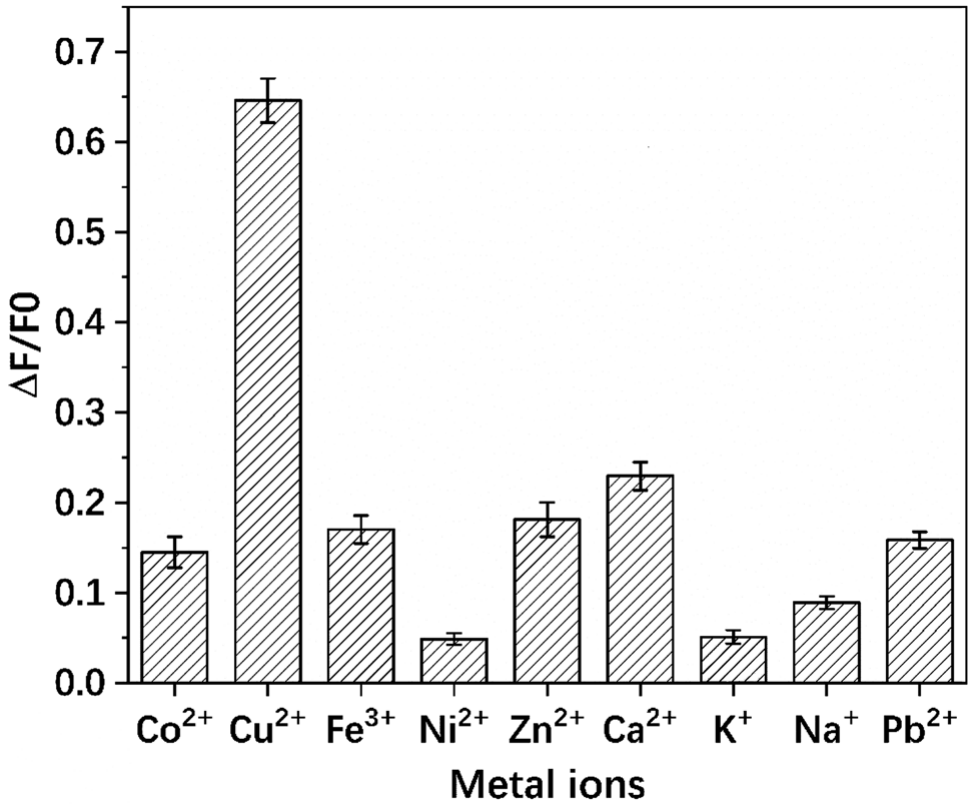
Fluorescence response of the CCYWDAHRDY-AuNCs upon the addition of various ions. The concentration of Cu^2+^ was 60 μmol/L, and the concentration of other metal ions was 600 μmol/L.

To evaluate the selectivity of CCYWDAHRDY-AuNCs determination system to Cu^2+^, the impact of other metal ions, *i.e.,* Co^2+^, Fe^3+^, Ni^2+^, Zn^2+^, Ca^2+^, K^+^, Na^+^, and Pb^2+^ on the fluorescence response was detected. As shown in Fig. 7, with the addition of other metal ions, the fluorescence of the CCYWDAHRDY-AuNCs did not significantly quenched, even the concentration of other interfering ions was 10 times of Cu^2+^. Therefore, the method has good sensitivity and selectivity. The prepared CCYWDAHRDY-AuNCs has good fluorescence and stability, so the repeatability of the test results could be guaranteed.

## Conclusion

In summary, CCYWDAHRDY sequence was designed and CCYWDAHRDY-AuNCs was successfully synthesized. The optimal synthesis conditions of pH was 6.0, reaction time was10 min, and calcination temperature was 30 °C. The CCYWDAHRDY-AuNCs showed high selectivity to Cu^2+^, and the minimum detection limit was 52 nmol/L, the fluorescence intensity of the Cu^2+^ and the CCYWDAHRDY-AuNCs was linear in the 0.1 ~ 4.2 μmol/L range. Compared with AuNCs, the detection of Cu^2+^ by CCYWDAHRDY-AuNCs was more sensitive with a high specificity. These results indicated that the synthesized CCYWDAHRDY-AuNCs could be used to detect the Cu^2+^.
